# Editorial: Computational modeling and simulation of quadrupedal animal movement

**DOI:** 10.3389/fbioe.2022.943553

**Published:** 2022-07-19

**Authors:** Gina Bertocci, John R. Hutchinson, Denis J. Marcellin-Little, Marcus G. Pandy

**Affiliations:** ^1^ Department of Bioengineering, University of Louisville, Louisville, KY, United States; ^2^ Department of Comparative Biomedical Sciences, The Royal Veterinary College, University of London, London, United Kingdom; ^3^ Department of Surgical and Radiological Sciences, University of California, Davis, Davis, CA, United States; ^4^ Department of Mechanical Engineering, University of Melbourne, Parkville, VIC, Australia

**Keywords:** modeling, simulation, dynamics, motion, animal, quadruped, biomechanics

Computational modeling and simulation used to study movement and function in healthy humans, as well as in those with clinical pathologies, is a well-developed field. Computational models of movement can provide objective insights into skeletal motion, muscle/ligament and joint loading, muscle activation patterns, coordination of multi-joint movement, injury mechanisms, compensatory locomotion, and other dynamic anatomical functions. Anatomically accurate models developed from medical imaging in combination with biomechanical properties can provide a window into *in vivo* function that would otherwise not be possible without subject experimentation or invasive testing. Modeling and simulation enable the investigation of the dynamic interaction between musculoskeletal and neuromuscular systems needed to generate motion and can be used to study pathologies, injury mechanisms, joint replacement/implants, rehabilitation, prosthetics/orthotics, and sports medicine. Additionally, the predictive capabilities of *validated* models can answer “what if” questions, and through sensitivity analysis can determine the influence of parameters on outcomes. Only recently has computational modeling been used to study animal movement and function. Such models seek to not only understand functional movement of healthy quadrupeds, but also to advance our understanding of clinical pathologies to improve animal health.

In this Research Topic, researchers from the fields of bioengineering, robotics, mathematics, veterinary medicine, comparative biology and zoology, and kinesiology have advanced our understanding of quadrupedal animal movement and function using various types of computational models. Manuscripts in this Research Topic describe a wide range of models including anatomical/morphological models to explore musculoskeletal function, musculoskeletal models developed from advanced XROMM and diceCT imaging technologies and used to describe locomotor stability and dynamics or to enhance locomotor performance, hydrodynamics models to investigate the influence of surface properties on impact loading of the forelimb, neuromuscular models to study afferent feedback in locomotion, multibody models to evaluate the effects of uncertainties on microscale motion simulations, evolutionary models to characterize muscle morphology, kinematic models to evaluate spinal motion, and models of adaptive motion to advance robotic design. These computational models have been applied to a diverse array of quadrupeds ranging from Chihuahuas to Labrador Retrievers, cats to fossil crocodile relatives, and rodents to horses. A brief description of these leading-edge manuscripts follows.

Three papers in the Research Topic focus on the area of pathology in quadrupeds. A paper by Schaub et al. describes 3D motion of the lower lumbar spine and pelvis in German Shepherd dogs at a walk and trot evaluated using cineradiography. Intervertebral motion was small overall, under 3° of rotation and under 2 mm of translation. Surprisingly, however, intervertebral motion varied among strides within a dog and among dogs. The paper by Schikowski et al. describes 3D atlantooccipital and atlantoaxial kinematic motion of the craniocervical junction in Chihuahuas compared to Labrador Retrievers at a walk, which was also evaluated using cineradiography. Maximal sagittal, lateral, and axial rotation at the atlantooccipital and atlantoaxial joints ranged from 16° to 30° and was slightly larger in Chihuahuas than Labrador Retrievers. Computational modeling can also be used to improve our understanding of injury mechanisms. The paper by Harrison et al. focused on a coupled biomechanical-smoothed particle hydrodynamics model for horse racing tracks and describes the development and validation of a 3D computational model of an equine hoof interacting with a dirt surface and a synthetic track. This model describes the interaction of track surface properties and limb loading, and has the potential to provide insight into distal limb injuries in horse racing.

Several studies in this Research Topic used musculoskeletal models to better understand muscle morphological measures during dynamic motion. Regnault et al. used anatomical, kinematic and dynamic analyses in experimentally manipulated echidna (monotreme mammal) cadaveric forelimbs, while simultaneously capturing estimates of muscle moment arms around major joints. They used the moment arms to estimate maximal muscle moments, and tested whether using moment arms alone vs. joint moments (dependent on muscle size) yielded different conclusions about overall limb mechanics. This approach helped to evaluate the 3D musculoskeletal model, showing which assumptions were reasonable vs. where detailed inputs might be needed. In another study, Löffler et al. also investigated mammalian muscle moment arms in 3D, of hindlimb gluteus medius, but in caviomorph rodents across a broad evolutionary span. They tested how locomotor adaptations (e.g., digging, climbing, and running) related to leverage, finding support for their hypotheses that climbing caviomorphs tended to have larger moment arms, whereas diggers had smaller ones. Furthermore, the authors reconstructed the evolution of locomotor styles, showing how running adaptation was ancestral, with climbing and digging evolving independently, their muscle leverages promoted by natural selection. To investigate the mechanics of walking in frogs, Collings et al. conducted a “Functional analysis of anuran pelvic and thigh anatomy using musculoskeletal modeling of the *Phlyctimantis maculatus.”* In this study, a 3D musculoskeletal model demonstrated that lateral pelvic rotation influenced muscle moment arm in most hindlimb muscles. Findings indicated that pelvic influence was dependent upon femoral angle, which can further alter muscle function.

Lumped-parameter, rigid body biomechanical models were also used to investigate locomotor performance in quadrupedal gait. Ding et al., studying jerboa rodents, combined experimental data on locomotor dynamics with a Spring-Loaded Inverted Pendulum model, applying optimal control theory in simulations predicting where gait transitions should occur. They successfully predicted these transitions across a wide speed range and discovered two different bipedal gaits that were previously undescribed, producing a total of five different locomotor modes. The simulations revealed two major limb-control mechanisms used, involving angular displacement of limbs in swing phase. Using a 2D model with simple springlike limbs, but adding a flexible back joint, Yamada et al. simulated how body center of mass position in horses influenced gait selection. Their simulations used Poincaré maps identifying periodic solutions corresponding to six gait patterns, optimizing gait stability across a range of speeds. They found that the “real” center of mass position corresponds to the optimum for the transverse galloping gait, as opposed to the other five gait patterns. Adachi et al. used a similar modeling approach, varying model geometry to produce a more short-necked (dog-like) vs. long-necked (horse-like) morphology, adopting a quasi-3D approach (e.g., torsional joint allowing body roll). They investigated which periodic solutions for trotting gaits were optimal in terms of stability across a Poincaré map for nine gait patterns. Three patterns were favored, but varying the asymmetry of the model improved stability, demonstrating how horse morphology and forelimb stiffness promote gait differences from trotting dogs. Kamimura et al. also used Poincaré maps to obtain periodic solutions characterizing galloping mechanics in the fastest land animal on Earth: cheetah. A 2D model with rigid torso segments actuated by massless spring-like legs showed that only one periodic solution properly identified the three most distinctive features of cheetah galloping–limited vertical displacements of the mass center, small pitching movements of the torso, and appreciable bending of the spine–which in turn enable these animals to maximize running speed. Polet and Hutchinson used a trajectory optimization approach and 2D model, applied to a different species: the quadrupedal extinct archosaur *Batrachotomus*, from the Triassic Period. Their simulations predicted at which speeds gait transitions should occur, with good success when applied to experimental data from dogs which supported this methodology. For the fossil, the simulations suggested that there were two gait transitions, with gaits unlike those of extant trotting crocodiles. Importantly, they compared their results with fossilized trackways from similar Triassic archosaurs, obtaining reasonable agreement. Various metrics have been proposed for quantifying mechanical work in quadrupedal locomotion. Pelot and Bertram evaluated the ability of three such measures–Net COM Work, Individual Limbs COM Work, and Limb Extension Work–to predict the four-beat walking strategy commonly observed in cursorial quadrupedal mammals. Applying each of these metrics as cost functions, they solved a series of parameter optimization problems to find that only Limb Extension Work coupled with a model of distributed body mass predicted a walking gait that matches the stereotypical quadruped pattern. The number of legs used to propel the body during locomotion has a decisive effect on the motion of the center of mass and gait stability. Using a Leg Force Interference Model, Wiehmann sought to explain why terrestrial animals change the number of propulsive legs as running speed increases. His analysis showed that as running speed increases, the magnitude of the ground reaction force decreases consonant with the number of legs propelling the body forward. However, one caveat of these findings is that the results do not appear to apply to bipedal locomotion.

Neuromuscular control and force feedback are critical during dynamic activities. Two studies in this Research Topic incorporated these elements into feline models investigating locomotion and landing following descent. Kim et al. included cat hindlimb muscles and a neural network into a neuromusculoskeletal modeling framework to test how feedback influences locomotor dynamics during walking and perturbation via stepping into a hole. The model included Hill-type muscles causing forces to have length- and velocity-dependence. Simulations then used optimization to characterize walking dynamics, and dynamical systems theory to investigate how perturbations were addressed. Their experimental vs. simulation results exhibited good agreement, illustrating how feedback rapidly changes dynamics to compensate for perturbations. Xu et al. employed a feline model to study adaptive strategies in landing following descents from different heights using experimental forelimb dynamic data, finite element analysis (FEA) and machine learning. The FE model demonstrated agreement with experimental data and indicated that maximal limb/bone stress occurs at the joints. Considering force feedback, they determined that with increasing ground reaction force, loading shifts progressively from the distal limb joint to the middle joint and up proximal joint. This information can be used in the design of bionic robots to increase service life of mechanical limbs and reduce wear by strengthening materials used in joints.

Sensitivity analyses are often performed using musculoskeletal models to evaluate the influence of various relevant parameters on outcome. Arroyave-Tobón et al. used 3D skeletal modeling applied to the entire body of seed-collecting ants to quantify locomotor kinematics and to determine the sensitivity of outcomes to marker positions. Their analysis combined specimen geometry with raw locomotor kinematics data, then integrated via modelled inverse kinematic analysis. Monte Carlo simulations showed that marker positions displayed more sensitivity than joint angle inputs. While ants use hexapedal rather than quadrupedal locomotion, the principles applied here are the same and may inspire cross-fertilization of ideas.

Together, these studies in this Research Topic demonstrate the wide array of computational modeling applications addressing research questions related to quadrupedal locomotion and dynamics. With increasing parallel computing and graphics processing capabilities, dynamic optimization of advanced anatomical models and more realistic simulation of motion can further advance our understanding of functional movement in quadrupedal animals in future studies.

